# Allergic Rhinitis Symptoms and Impact Assessment (ARSIA) of Public Community in Saudi Arabia: A National Cross-Sectional Study

**DOI:** 10.7759/cureus.76423

**Published:** 2024-12-26

**Authors:** Wafaa Mohammad, Ali Alnajim, Rola H Alseghair, Bayan B Altowairqi, Layan B Alharbi, Yasmeen A Alhababi, Murtada Alqattan, Shatha Alsuwaida, Nouf M Alsubaie, Reem M Alfaqih

**Affiliations:** 1 College of Medicine, King Faisal University, Alahsa, SAU; 2 Collage of Medicine, King Faisal University, Alahsa, SAU; 3 College of Medicine, Qassim University, Qassim, SAU; 4 College of Medicine, Taif University, Taif, SAU; 5 College of Medicine, Imam Abdulrahman Bin Faisal University, Alahsa, SAU; 6 Faculty of Medicine, King Abdulaziz University, Jeddah, SAU; 7 Medicine, Salmaniya Medical Complex, Manama, BHR; 8 General Practice, Al-Qunfudha General Hospital, Al-Qunfudha, SAU

**Keywords:** allergic rhinitis (ar), allergic rhinitis severity, environmental allergen, prevalence of allergic rhinitis, socioeconomic impact

## Abstract

Introduction

Allergic rhinitis (AR) is a common inflammatory condition of the nasal mucosa in Saudi Arabia, triggered by various allergens. It significantly impacts daily life, affecting sleep quality, social interactions, and productivity. Despite its prevalence, AR is often underdiagnosed and undertreated in the region.

Methods

This national cross-sectional study was conducted in February 2024 using an online survey distributed via social media and Google Forms. Participants were Saudi Arabian residents with self-reported AR. The allergic rhinitis symptoms and impact assessment (ARSIA) questionnaire was used and translated into Arabic. A total of 262 participants completed the survey. Data were analyzed using SPSS, with descriptive statistics and chi-square tests to explore relationships between variables.

Results

The study included 262 participants, with a nearly balanced gender representation of 133 (50.8%) females and 129 (49.2%) males and a predominant age group of >30 years with 140 individuals (53.4%). The majority were Saudi nationals, 244 (93.1%). Educational levels varied, with undergraduates being the largest group of 132 (50.4%) respondents. Concerning smoking, 189 respondents (72.1%) never smoked. A high prevalence of allergen sensitivity was noted, with smoke (98.1%) and medications (99.2%) being the most common triggers. Medication use varied, with 114 (43.5%) participants not using any medication. Continuous symptoms were reported by 140 (53.4%) of participants, and 161 participants (61.5%) classified their symptoms as moderate to severe. Significant differences were observed based on nationality, education level, and social status concerning symptom severity.

Conclusion

AR significantly impacts the Saudi Arabian population, with high allergen sensitivity and varied medication use. The study underscores the need for improved diagnosis and treatment strategies, considering the unique environmental and demographic factors in the region. Further research is necessary to develop effective interventions tailored to the local context.

## Introduction

Allergic rhinitis (AR) is a prevalent inflammatory condition affecting the nasal mucosa in Saudi Arabia, triggered by various indoor and outdoor allergens. Symptoms include nasal congestion, rhinorrhea, itching, sneezing, and ocular issues such as redness and tearing [1]. Globally, allergic diseases affect approximately 10-30% of adults and up to 40% of children, yet AR is often underdiagnosed and undertreated, particularly in Saudi Arabia [2].

Local studies point to a significant gap in diagnosis; for example, in Saudi Arabia's western region, 24% of participants displayed symptoms of AR, but only 4% had received a formal diagnosis [3]. The impact of AR goes beyond physical discomfort; it affects social interactions, academic and work performance, and significantly reduces sleep quality [2,4].

In a related trend, children in Saudi Arabia experience rhino-conjunctivitis at a rate below the global average. However, the prevalence among adolescents aligns with the global average, yet the occurrence of severe cases in this group is notably double the global average [5]. This highlights the varying severity and prevalence of related allergic conditions across different age groups within the region.

Environmental factors unique to Saudi Arabia, such as frequent sandstorms, exacerbate AR symptoms by dispersing various allergens, thereby worsening respiratory conditions [6]. This emphasizes the need for region-specific research and intervention strategies.

Treatment methods in Saudi Arabia include standard pharmacological approaches like antihistamines and corticosteroids, as well as allergen immunotherapy and various culturally influenced non-pharmacological methods [1]. These alternative strategies often draw from cultural traditions and personal experiences rather than being based on scientific evidence [7].

The significant burden of AR on Saudi society and the varied treatment practices highlight the critical need for comprehensive local studies. These studies should investigate the epidemiology and clinical features of AR and evaluate the effectiveness of both traditional and non-traditional treatments [8,9]. Consequently, this study aims to provide a detailed national cross-sectional evaluation of allergic rhinitis symptoms and impact in Saudi Arabia.

## Materials and methods

This study was a cross-sectional survey carried out in February 2024 across different areas of Saudi Arabia using a convenience sampling approach. It aimed to provide allergic rhinitis symptoms and impact in Saudi Arabia, utilizing an online survey distributed via social media and Google Forms (Google, Inc., Mountain View, US).

Eligibility criteria

The research focused on Saudi Arabian residents who self-reported having allergic rhinitis. Exclusion criteria included individuals who did not fit the study profile, those who did not complete the survey, or those who opted out of participation and thus were excluded from subsequent analysis.

Assessment instrument

The research utilized allergic rhinitis symptoms and impact assessment (ARSIA) questionnaire [10], translated into Arabic. This questionnaire included three main parts: (A) six questions related to participant demographics, (B) ten multiple-choice questions aimed at evaluating the course of the disease as intermittent or continuous, and (C) seven multiple-choice questions evaluating the severity and impact of allergic rhinitis. A pilot study with 20 participants was conducted to test the reliability of the Arabic version, which demonstrated a Cronbach alpha of 0.797, indicating high internal consistency suitable for this population.

A detailed scoring system was implemented to categorize participants based on their responses. Symptoms were scored based on severity, where 0 indicated no symptoms, 1 indicated mild symptoms, 2 indicated moderate symptoms, and 3 indicated severe symptoms. Additionally, the frequency of symptoms was scored with 0 for never, 1 for occasionally (1-4 times per week), 2 for frequently (5-6 times per week), and 3 for always (daily). The total score for each participant was used to classify the severity and impact of allergic rhinitis. Specifically, in the nasal symptoms domain, a score of 12 or less indicated intermittent symptoms, while a score above 12 indicated persistent symptoms. For the domain of impact on daily activities, a cut-off of 15 points was used - scores of 15 or less were classified as mild, whereas scores above 15 were classified as moderate to severe. This classification system allowed for a nuanced assessment of both the frequency and severity of symptoms, aligning with Allergic Rhinitis and its Impact on Asthma (ARIA) guidelines.

Sample size

The minimum sample size was calculated using the single proportion sample size formula, assuming a 50% proportion, 95% confidence level, and 5% margin of error. This calculation suggested a minimum requirement of 385 participants.

Data analysis

Data were processed and analyzed using SPSS (IBM, Inc., Armonk, US). Descriptive statistics were used to determine means, medians, modes, standard deviations, and frequencies, while relationships between variables were explored using chi-square tests, with a significance threshold set at a p-value of 0.05.

Ethical considerations

Ethical approval was obtained from the ethical committee of the Deanship of Scientific Research at King Faisal University, under reference number KFU-REC-2024-MAY-ETHICS2429. Participation was entirely voluntary, with participants fully briefed on the study's aims and the time commitment involved. Informed consent was obtained electronically before participation.

## Results

The study included 262 participants who self-reported allergic rhinitis. As shown in Table 1, age distribution shows a predominant group with participants age from 18 to 30 years, comprising 110 individuals (42.0%), followed by those over 30 years, with 140 individuals (53.4%), and a smaller group under 18 years, totaling 12 individuals (4.6%). Gender representation is nearly balanced, with 133 females (50.8%) and 129 males (49.2%). The vast majority of participants are Saudi nationals, accounting for 244 individuals (93.1%), compared to 18 non-Saudi participants (6.9%).

**Table 1:** Sociodemographic profile of the study participants

Sociodemographic factors		n	%
Age	<18	12	4.6%
	18-30	110	42.0%
	>30	140	53.4%
Gender	Male	129	49.2%
	Female	133	50.8%
Nationality	Saudi	244	93.1%
	Non-Saudi	18	6.9%
Education	Uneducated	2	0.8%
	Elementary school	10	3.8%
	Middle school	23	8.8%
	High school	88	33.6%
	Undergraduate	132	50.4%
	Postgraduate	7	2.7%
Social status	Married	136	51.9%
	Single	105	40.1%
	Divorced	16	6.1%
	Widow	5	1.9%
Smoking status	Current	49	18.7%
	Never	189	72.1%
	Ex-smoker	24	9.2%

Educational levels among participants varied, with the largest group being undergraduates, with 132 respondents (50.4%). This is followed by those with a high school education, 88 respondents (33.6%). Only a small number, seven participants (2.7%), reported having postgraduate education.

Regarding social status, the majority were married, 136 (51.9%), and 105 were single (40.1%). The remaining participants were either divorced, 16 (6.1%), or 5 (1.9%) widowed. Regarding smoking status, the majority of participants never smoked, 189 (72.1%), while 49 were current smokers (18.7%), and 24 were ex-smokers (9.2%).

Table 2 provides a detailed breakdown of the allergens and medications associated with allergic rhinitis among the study population. The prevalence of allergen sensitivity was notably high, with 257 respondents (98.1%) affected by smoke and 260 respondents (99.2%) by medications, representing the most common triggers. Other significant allergens included food, affecting 256 respondents (97.7%), and animals, affecting 253 respondents (96.6%). Dust and weather changes also contributed to allergic reactions in 217 respondents (82.8%) and 232 (88.5%), respectively.

**Table 2:** Allergens and medications profile across the study participants

Allergic rhinitis profile		n	%
Allergens	Food	256	97.7%
	Dust	217	82.8%
	Animals	253	96.6%
	Whether changes	232	88.5%
	Smoke	257	98.1%
	Medications	260	99.2%
Medications	Anti-histamines	50	19.1%
	Antiallergic sprays	61	23.3%
	Both	37	14.1%
	No medications	114	43.5%

Regarding medication use, a substantial proportion of participants did not use any medications to manage their symptoms, accounting for 114 respondents (43.5%). In contrast, 61 participants (23.3%) used antiallergic sprays, and 50 participants (19.1%) used antihistamines. A smaller group of 37 respondents (14.1%) reported using both types of medications to manage their condition.

The classification of allergic rhinitis by course and severity is detailed in Table 3 and exhibits varied patterns among participants. A slightly higher percentage of respondents reported continuous symptoms, 140 (53.4%), compared to those with intermittent symptoms, 122 (46.6%). Regarding severity, most participants classified their symptoms as moderate to severe, which included 161 respondents (61.5%). In contrast, those reporting mild symptoms comprised 101 respondents (38.5%).

**Table 3:** Classification of allergic rhinitis by course and severity

Classification of the course and severity of allergic rhinitis		n	%
Course	Intermittent	122	46.6%
	Continuous	140	53.4%
Severity	Mild	101	38.5%
	Moderate/severe	161	61.5%

Significant differences were observed among the participants concerning nationality, education level, and social status. Specifically, non-Saudi participants reported fewer moderate or severe conditions compared to Saudi participants (p=0.002). Additionally, individuals with only middle school education were more likely to experience moderate to severe conditions (p=0.003). Concerning social status, divorced individuals predominantly reported mild conditions, marking a significant deviation (p=0.0001; see Table 4).

**Table 4:** Association of sociodemographic characteristics to the course and severity of allergic rhinitis * p<0.05 using χ2 test

Variables		Course		p-value	Chi-square value	Severity		p-value	Chi-square value
		Intermittent	Continuous			Mild	Moderate/severe		
Age	<18	5	7	0.873	0.272	8	4	0.089	4.847
	18-30	53	57			44	66		
	>30	64	76			49	91		
Gender	Male	61	68	0.818	0.053	51	78	0.747	0.104
	Female	61	72			50	83		
Nationality	Saudi	115	129	0.499	0.458	88	156	0.002*	9.251
	Non-Saudi	7	11			13	5		
Education	Uneducated	0	2	0.729	2.809	2	0	0.003*	18.209
	Elementary school	5	5			5	5		
	Middle school	9	14			17	6		
	High school	43	45			29	59		
	Undergraduate	61	71			45	87		
	Postgraduate	4	3			3	4		
Social status	Married	63	73	0.980	0.188	45	91	0.0001*	21.126
	Single	50	55			42	63		
	Divorced	7	9			14	2		
	Widow	2	3			0	5		
Smoking status	Current	25	24	0.786	0.482	19	30	0.106	4.494
	Never	86	103			68	121		
	Ex-smoker	11	13			14	10		

Conversely, the analysis showed no significant differences in the course or severity of the condition across age groups, gender, or smoking status. The uniformity in distribution suggests that these factors did not significantly influence the condition's severity or progression. The results from Table 4 thus provide a detailed view of the demographic influences on the condition studied, reflecting variability in severity linked with specific demographic factors without suggesting a causative relationship.

## Discussion

Allergic rhinitis remains a prevalent yet often overlooked condition in Saudi Arabia, causing substantial disruption to daily activities due to a range of symptoms, from nasal congestion to ocular irritation. Given its significant but frequently under-recognized impact on the population, there is a critical need for targeted research to advance diagnosis and treatment practices across the region [1-2]. The data reveals a strikingly high sensitivity among participants to common allergens, with smoke and medications being the most prevalent triggers, affecting 257 (98.1%) and 260 (99.2%) participants, respectively. These sensitivities are possibly exacerbated by environmental factors such as pollution and prevalent smoking habits, underscoring the need for public health interventions to address these issues [11].

Furthermore, the high rates of sensitivity to other allergens, such as food (97.7%), animals (96.6%), weather changes (88.5%), and dust (82.8%), indicate that dietary factors, pet contact, and the arid, dusty climate of Saudi Arabia significantly contribute to the incidence of allergic rhinitis. Despite these high rates of allergen sensitivity, a substantial 114 (43.5%) of participants do not use any medications to manage their symptoms, which may reflect underdiagnosis, limited healthcare access, or reluctance to use pharmaceutical treatments [6,11]. Among those who do use medication, the predominant reliance on antiallergic sprays with 61 individuals (23.3%) and antihistamines with 50 individuals (19.1%) suggests a need for better public education and healthcare strategies to improve understanding and management of the disease [4].

Our study's findings highlight the classification of allergic rhinitis by course and severity among participants. A higher incidence of continuous symptoms representing 140 individuals (53.4%) compared to intermittent symptoms with 122 individuals (46.6%) suggests persistent exposure to allergens or the presence of more severe underlying allergic responses. This prevalence of continuous symptoms aligns with findings from the International Study of Asthma and Allergies in Childhood (ISAAC), indicating that urbanization increases the risk of continuous allergic rhinitis due to constant allergen exposure [1]. Greiner et al. also demonstrated that continuous exposure to allergens, especially in urban settings, leads to chronic manifestations of allergic rhinitis [12].

Furthermore, the data shows that 161 (61.5%) of participants experience moderate to severe symptoms, underscoring the substantial impact of allergic rhinitis on individuals' health and quality of life. This significant health burden reflects the global trend where moderate to severe allergic rhinitis is often associated with greater healthcare use and reduced quality of life. The prevalence of severe symptoms may indicate gaps in effective allergy management and prevention strategies, as well as the need for enhanced diagnostic, therapeutic, and educational strategies to manage allergic rhinitis effectively [3]. This severity distribution necessitates tailored public health initiatives and individualized treatment approaches, particularly in regions with high pollution levels or specific allergen prevalence.

With regard to environmental and lifestyle factors contributing to this trend, studies by Zuberbier et al. have linked urbanization and changes in lifestyle to increased allergic rhinitis severity, emphasizing the economic burden of inadequate management of allergic diseases [13]. Brożek et al. also recommend integrated care pathways for multi-morbid respiratory diseases, including allergic rhinitis, to improve overall patient well-being and reduce the risk of severe asthma [14].

One of the fundamental aspects of the study is to evaluate the impact of the demographic characteristics of participants on the course and severity of allergic rhinitis among the Saudi population, a nationwide study. The current study revealed a significant association between the nationality of the participants and the severity of the allergic rhinitis symptoms, with most of the respondents being Saudis with moderate to severe symptoms. On the other hand, the non-Saudi reported a mild form of the disease. Similarly, a cross-sectional study performed in Saudi Arabia in 2018 concluded a significant prevalence of allergic rhinitis with severe symptomatology among Saudis [1]. However, some authors believe that it is possible to observe various results in the context of the prevalence and severity within the same region. A cross-sectional study conducted in the western region of Saudi Arabia (Jeddah) found a lower prevalence of allergic rhinitis (37%). Moreover, a meta-analysis conducted in 2022 concluded that the overall prevalence of allergic rhinitis among the Saudi population is 21.2% [15,16]. Both latter studies showed lower prevalence, which can be attributed to sample size, the chosen regions with a variety of triggers and risk factors, and the use of different assessment questionaries compared to the present study. Also, this study reported a positive correlation between higher levels of education and severity of allergic rhinitis. The majority of participants were represented undergraduates with severe symptoms of allergic rhinitis (33.2%), which is consistent with the findings of the cross-sectional study done in Jeddah; therefore, the higher rates are believed to be due to stress [15]. Furthermore, the study found a significant relationship between marital status and the severity of allergic rhinitis symptoms (p=0.0001). Married participants were more likely to report moderate to severe symptoms in 91 respondents compared to mild symptoms in 45 respondents, potentially due to added stress and shared environmental factors that increase allergen exposure. This aligns with El-Gamal et al. [15], which identified stress as an exacerbating factor for allergic rhinitis severity. Single participants showed a more balanced distribution of symptom severity, while divorced participants predominantly reported mild symptoms, possibly due to more control over their environment. AlShehri et al. [17] similarly noted that changes in lifestyle after marital transitions can impact allergic outcomes. Similarly, lifestyle changes after marital transitions can impact allergic outcomes. However, AlShehri et al. [17] found that a substantial percentage of married participants reported mild symptoms of allergic rhinitis, suggesting that the impact of marital status on symptom severity may vary depending on lifestyle or regional differences. The variations between the current study and the last study are explained by the differences in the size of the sample, the geographical regions where the study was done, and the type of allergens and sensitizers.

Limitations

This study has several limitations. The convenience sampling method and the use of an online survey may limit the generalizability of the findings to the broader Saudi population, as it might exclude certain demographics, such as older adults or those with limited internet access. Additionally, the reliance on self-reported data for AR symptoms and diagnosis may introduce a recall bias or inaccuracies in participant responses. Despite these limitations, the study provides valuable insights into the prevalence and impact of allergic rhinitis in Saudi Arabia and underscores the importance of region-specific research for effective intervention strategies.

## Conclusions

This national cross-sectional study assessed allergic rhinitis symptoms and their impact on the Saudi Arabian population. Out of the total of 262 participants, the majority reported high sensitivity to common allergens, particularly to smoke, with 257 respondents (98.1%), and medications, with 260 respondents (99.2%). The study highlighted significant demographic associations, including that Saudi participants were more likely to report moderate to severe symptoms compared to non-Saudis. Additionally, participants with only middle school education had a higher likelihood of experiencing severe symptoms. Continuous symptoms were reported by 140 participants (53.4%), and 161 (61.5%) classified their symptoms as moderate to severe, underscoring the substantial health burden of AR. Furthermore, a notable portion of the study population, 114 (43.5%), did not use any medication for AR, reflecting potential underdiagnosis or a lack of access to treatment. The findings emphasize a critical need for improved public health initiatives, including widespread awareness campaigns, targeted screening programs, and accessible treatment options tailored to the unique environmental and demographic factors of the region. Efforts should also focus on addressing gaps in medication use and advancing diagnostic approaches to enhance disease management. By prioritizing these strategies, healthcare providers can alleviate the personal and societal impact of AR, contributing to better overall health outcomes in Saudi Arabia.
